# Acute Toxicity and Early Oncological Outcomes After Intraoperative Electron Radiotherapy (IOERT) as Boost Followed by Whole Breast Irradiation in 157 Early Stage Breast Cancer Patients—First Clinical Results From a Single Center

**DOI:** 10.3389/fonc.2019.00384

**Published:** 2019-05-21

**Authors:** Laila König, Kristin Lang, Jörg Heil, Michael Golatta, Gerald Major, David Krug, Juliane Hörner-Rieber, Matthias F. Häfner, Stefan A. Koerber, Semi Harrabi, Tilman Bostel, Jürgen Debus, Matthias Uhl

**Affiliations:** ^1^Department of Radiation Oncology, University Hospital Heidelberg, Heidelberg, Germany; ^2^Heidelberg Institute of Radiation Oncology (HIRO), Heidelberg, Germany; ^3^Department of Gynecology and Obstetrics, University Hospital Heidelberg, Heidelberg, Germany; ^4^Department of Radiation Oncology, University Hospital Schleswig Holstein, Kiel, Germany; ^5^Department of Radiation Oncology, University Hospital Mainz, Mainz, Germany

**Keywords:** breast cancer, boost radiotherapy, electrons, intraoperative radiotherapy, IOERT, IORT

## Abstract

**Introduction:** Breast conserving surgery (BCS) followed by postoperative whole breast irradiation (WBI) is the current standard of care for early stage breast cancer patients. Boost to the tumor bed is recommended for patients with a higher risk of local recurrence and may be applied with different techniques. Intraoperative electron radiotherapy (IOERT) offers several advantages compared to other techniques, like direct visualization of the tumor bed, better skin sparing, less inter- and intrafractional motion, but also radiobiological effects may be beneficial. Objective of this retrospective analysis of IOERT as boost in breast cancer patients was to assess acute toxicity and early oncological outcomes.

**Material and Methods:** All patients, who have been irradiated between 11/2014 and 01/2018 with IOERT during BCS were analyzed. IOERT was applied using the mobile linear accelerator Mobetron with a total dose of 10 Gy, prescribed to the 90% isodose. After ensured woundhealing, WBI followed with normofractionated or hypofractionated regimens. Patient reports, including diagnostic examinations and toxicity were analyzed after surgery and 6–8 weeks after WBI. Overall survival, distant progression-free survival, in-breast and contralateral breast local progression-free survival were calculated using the Kaplan-Meier method. Furthermore, recurrence patterns were assessed.

**Results:** In total, 157 patients with a median age of 57 years were evaluated. Postoperative adverse events were mild with seroma and hematoma grade 1–2 in 26% and grade 3 in 0.6% of the patients. Wound infections grade 2–3 occurred in 2.2% and wound dehiscence grade 1–2 in 1.9% of the patients. Six to eight weeks after WBI radiotherapy-dependent acute dermatitis grade 1–2 was most common in 90.9% of the patients. Only 4.6% of the patients suffered from dermatitis grade 3. No grade 4 toxicities were documented after surgery or WBI. 2- and 3-year overall survival and distant progression-free survival, were 97.5 and 93.6, and 0.7 and 2.8%, respectively. In-breast recurrence and contralateral breast cancer rates after 3 years were 1.9 and 2.8%, respectively.

**Conclusion:** IOERT boost during BCS is a safe treatment option with low acute toxicity. Short-term recurrence rates are comparable to previously published data and emphasize, that IOERT as boost is an effective treatment.

## Introduction

Breast conserving surgery (BCS) followed by postoperative whole breast irradiation (WBI) is the current standard of care for early stage breast cancer patients. Several randomized trials have demonstrated, that postoperative WBI shows a significant benefit regarding the oncological outcome by reducing recurrence rates and breast cancer death (1, 2). WBI can either be applied normofractionated with a dose of 50 Gy (e.g., in 25–28 fractions) or more commonly during the last years with a hypofractionated scheme of 40 Gy (e.g., in 15–16 fractions) (3–5).

In selected patients, the tumor bed itself represents a region with higher probability of in-breast recurrence, thus an additional boost dose of 10–16 Gy (5–8 × 2 Gy) significantly reduces local recurrence rates (6–8) and is therefore recommended for younger patients or for patients with other risk factors related to a higher risk of local recurrence. For boost application, different techniques are available, including external beam radiotherapy (EBRT) brachytherapy or intraoperative radiotherapy (kV-IORT or electron-IORT) (6, 7, 9–11).

Some concerns exist regarding boost application with EBRT: Due to increasing rates of oncoplastic surgery, there is a substantial risk for geographical miss of the tumor bed by using EBRT after surgery. Furthermore, EBRT yields in a higher skin dose, which may lead to higher rates of fibrosis and therefore worse cosmetic results (12).

In contrast to EBRT, IORT allows a precise intraoperative irradiation of the area with the highest risk for subclinical cell contamination (tumor bed) before the oncoplastic reconstruction takes place. Additionally, due to the high single dose, intra- and interfractional motion is reduced and dose will be applied more homogeneously. Overlying skin is not exposed to radiation resulting in better skin sparing. Since IORT is applied in a single dose during BCS, this technique offers a higher patient comfort and a reduction of the total treatment time by 1–2 weeks (13). Finally, there are also radiobiological advantages: The lower alpha/beta ratio (~4) results in higher sensitivity against higher doses and an IORT dose of 10 Gy amounts to a BED of 35 Gy, being isoeffective to an EQD2 of 24 Gy (14). Furthermore, possible repopulation of residual tumor cells between surgery and EBRT is prevented and IORT could offer the possibility of a higher oxygenation status, which are subject of ongoing research. Besides, a role in abrogating the proliferative cascade induced by surgical wound healing is discussed and has been described *in vitro* (15–17). The largest patient cohort treated with electron IORT (IOERT) as boost was provided by a collaborative analysis within the European Group of the International Society of Intraoperative Radiotherapy (ISIORT). The analysis evaluated 1,109 patients treated at 7 different centers with a median follow up of 72 months. Local tumor control rates were excellent with 99.2% and an annual in-breast recurrence rate of 0.64, 0.34, 0.21, and 0.16% in patients <40 years, 40–49 years, 50–59 years, and ≥60 years, respectively (9). To compare our results of IOERT as boost radiotherapy in breast cancer patients receiving BCS, we conducted a monocentric, retrospective analysis of all patients, who were treated with IOERT at the University Hospital Heidelberg.

## Materials and Methods

### Patient Selection and Preoperative Treatment

From 11/2014 until 01/2018, 157 patients were irradiated with IOERT during BCS at the Department of Radiation Oncology at the University Hospital of Heidelberg.

Patients were eligible for IOERT if they had an early stage breast cancer (cT1-T2 cN0-1) suitable for breast conserving surgery, followed by adjuvant irradiation (whole breast irradiation and boost) with indication for boost radiotherapy. According to the current guideline (18), neoadjuvant chemotherapy was indicated as follows: for patients with Luminal B [HER-2 positive or Her2-negativ with a high proliferation index (≥40% ki67 or G3)], nodal positive (N+) or triple-negative tumors. For patients with ki67 11–39% and G2 tumors an additional genetic testing was indicated and in case of a high recurrence score, NACT was recommended by the treating gynecooncologist.

### Surgery and Radiotherapy

Lumpectomy with sentinel node or axillary lymph node dissection was performed according to the current guidelines or study protocols (18). Frozen sections to ensure tumor-free margins were performed before IOERT.

Prior to IOERT, the tissue surrounding the tumor bed was temporarily approximated by sutures according to the publication of the ISIORT pooled analysis (9).

The clinical target volume was decided according to the site and size of the tumor. We used a 1 cm margin in all directions and tubes were chosen accordingly. According to breast size and surgical wound/access tube size was adapted. For patients with complete response after NACT we used at least a tube with a 3 cm diameter.

**Figure 1:**
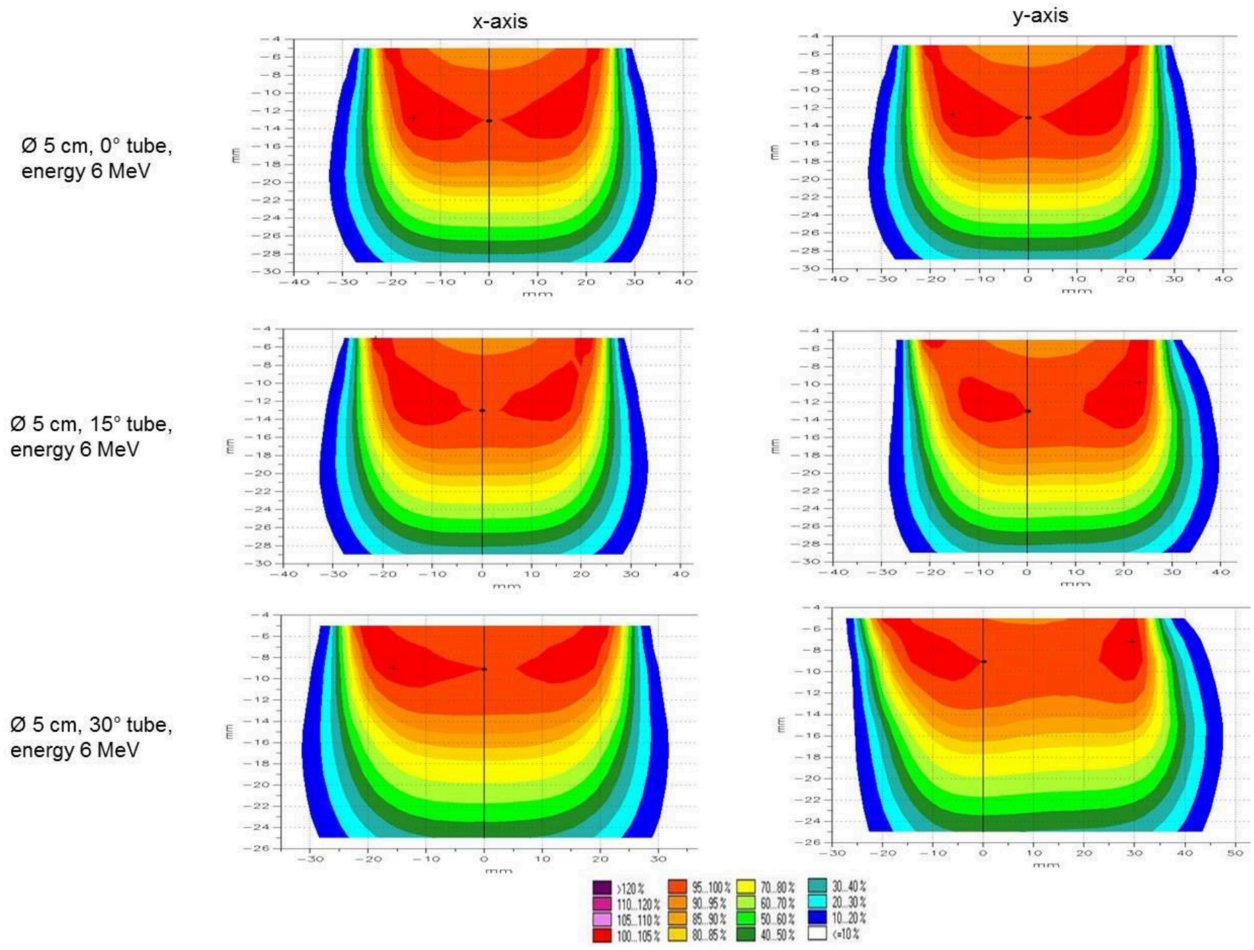
Isodose curves. Example of isodose distribution of 5 cm applicator tube with 0, 15, and 30° tube angle measured for 6 MeV beams.

The Mobetron® (IntraOP Medical), a self-shielding, mobile linear accelerator, was used to deliver IOERT. Energy of electron beams ranged from 6, 9 to 12 MeV, and was chosen according to the depth of the tumor bed which was measured with a ruler before IOERT. We prescribed a total dose of 10 Gy to the 90% isodose. The used tubes ranged from 3 to 6 cm in diameter, available in 0.5 cm steps and with angles of 0, 15, or 30°. An example of dose distribution is shown in Figure 1. After ensured woundhealing, WBI followed with either normofractionated or hypofractionated regimens. For patients with normal anatomy and right-sided breast cancer, WBI was performed with 3D-conformal radiotherapy. In patients with left-sided breast cancer or expected higher lung doses due to anatomical variations, WBI was performed with VMAT (volumetric modulated arc therapy) and DIBH (deep inspirational breath hold technique) technique. Align RT System was used for intra-fraction real time monitoring. Energies between 6 and 18 MV were chosen according to breast size and anatomy, so that dose prescription according to the ICRU recommendations 50 and 62 were adhered to.

### Follow-Up and Toxicity

Follow-up consisted of regular checkups according to international breast cancer guidelines with mammograms and clinical examinations (18).

Toxicity was scored after operation (acute postoperative toxicity after IOERT) and 6–8 weeks after WBI (acute toxicity after WBI) and was described according to the Common Terminology Criteria for Adverse Events (CTCAE) criteria (version 3.0) by reviewing the patient charts.

Acute toxicity was analyzed from clinical examinations of the gynecologists and radiation oncologist during the postoperative checkups, radiotherapy visits and follow-up visits. Additionally, we reviewed breast sonography scans as well as radiotherapy planning CT-scans to collect information about postoperative seroma and hematoma.

Follow up visits were done after operation and before WBI by the gynecologist and radiation oncologist, 6–8 weeks after WBI by the radiation oncologist and further follow ups according to the German guideline in regular intervals with clinical examinations every 3 months and breast sonographies every 6 months for the first 3 years and yearly mammograms during the whole follow up (18).

### Overall Survival (OS) and Time to Progression (TTP)

The follow up time was defined from the date of IOERT until death (for OS) or last known follow up. Time to progression represents the length of time from the date of IOERT to disease progression. Every known death (for OS), locoregional (for ibLPFS and cbPFS) or distant tumor progression (for DPFS) were counted as events. Patients alive were counted as censored.

### Statistical Analysis

For statistical analysis Kaplan-Meier estimates were used to calculate OS, distant progression-free survival (DPFS), in-breast local progression-free survival (ibLPFS) and contralateral breast cancer progression-free survival (cbPFS). These were conducted using IBM SPSS software version 24. Due to the small amount of recurrences, subgroup analyses were not reasonable.

### Ethics

This study was performed following institutional guidelines and the Declaration of Helsinki of 1975 in its most recent version. This study was approved by the Independent local Ethics Committee Heidelberg (Ref. Nr. S-164/2012).

## Results

### Patients and Tumor Characteristics

In total, 157 female patients with a median age of 57 years (range: 27–74 years) have been treated with IOERT boost during BCS from November 2014 until January 2018. The median follow-up was 24 months (range: 9–43 months) for all patients.

Most of the patients had early stage breast cancer (72% cT1, 28% cT2, 92% cN0, 8% cN1) with Luminal A phenotype (64%). Complete patient and tumor characteristics are displayed in Table 1.

**Table 1:** Patient and tumor characteristics.

**Total 157 patients**	***n* (%)**
Median age in years (range)	57 (29–74)
**Side**
Right	85 (53.8)
Left	72 (45.6)
**T stage**	cT	pT
T0 (ypT0)	–	7 (4.5)
T1a	16 (10.2)	25 (15.9)
T1b	30 (19.1)	33 (21.0)
T1c	67 (42.7)	65 (41.4)
T2	44 (28.0)	27 (17.2)
**N stage**	cN	pN
N0	145 (92.4)	128 (81.5)
N1	12 (7.6)	28 (17.8)
N2	0 (0.0)	1 (0.6)
**Grading**	Biopsy	Specimen
G1	47 (29.9)	42 (26.8)
G2	80 (51.0)	88 (56.1)
G3	30 (19.1)	20 (12.7)
n.a.	–	7 (4.5)
**Immunophenotype**
Luminal A	101 (64.3)
Luminal B	29 (18.5)
TNBC	15 (9.6)
Her2	12 (7.6)

*TNBC, triple negative breast cancer*.

A difference between pathological review in the biopsy compared to the operation specimen was documented in 75/157 patients (47.8%):

Upstaging regarding the T-status occurred in 25 patients (15.9%), of whom 2 (1.3%) had NACT. Downstaging was seen in 50 (31.8%) patients, of whom 31 (19.7%) patients had NACT. Eighty-two patients (52.2%), of whom 6 patients (3.8%) had NACT, had no change of T-status.

Upstaging regarding the N-status was observed in 24 (15.3%) patients, of whom 4 (2.5%) had NACT. Downstaging occurred in 6 (3.8%) patients, of whom 5 (3.2%) patients had NACT. No stage shift was seen in 127 patients (80.9%), of whom 30 patients (19.1%) had NACT.

### Treatment Characteristics

In the majority of the patients, sentinel lymph node dissection (87%) was performed. Twelve percent of the patients received axillary lymph node dissection and 2 patients with cN0 did not receive any lymph node dissection, since they were included in the INSEMA study (19, 20). A re-excision due to positive margins in the final histopathological section compared to the intraoperative frozen section was needed in 18 patients (11.5%).

Thirty-nine patients (25%) received neoadjuvant and 9 patients (6%) adjuvant chemotherapy. Seven out of thirty nine patients receiving NACT had a pathological complete remission (pCR, ypT0 ypN0) in the operation specimen.

WBI followed after a median time of 52 (range 12–305) days after surgery and was applied with a normofractionated scheme in 89% of the patients, and with a hypofractionated scheme in 9% of the patients. Four patients (2.5%) did not receive WBI: 1 patient developed a meningeosis carcinomatosa shortly after operation, so that systemic therapy was indicated and 3 patients (2%) refused further WBI. During follow up, no local recurrence was detected in the 3 patients who refused WBI. Detailed treatment characteristics are shown in Table 2.

**Table 2:** Treatment characteristics.

**Total 157 patients**	***n* (%)**
SLND	134 (85.4)
ALND	21 (13.4)
No LN dissection/sampling	2 (1.3)
Re-resection needed due to R+ status	18 (11.5)
Neoadjuvant chemotherapy	39 (24.8)
Adjuvant chemotherapy	9 (5.7)
Adjuvant hormonal therapy	142 (90.4)
**WBI**
Hypofractionated (40,05 Gy in 15 fractions)	14 (8.9)
Normofractionated (50/50,4 G in 25/28 fractions)	139 (88.5)
WBI not applied	4 (2.5)
Median time (days) from OP to WBI (range)	52 (12–305)

*SLNE, sentinel lymph node dissection; ALDN, axillary lymph node dissection; LN, lymph node; WBI, Whole breast irradiation*.

### Toxicity

Acute postoperative toxicity after IOERT was evaluable for all patients; acute toxicity 6–8 weeks after WBI for 153, since 4 patients did not receive WBI.

Most of the adverse events after surgery were mild according to the CTCAE v. 3.0. After revision of planning CT scans and ultrasound examinations, seroma and hematoma grade 1 (asymptomatic) and 2 (simple aspiration needed) occurred in 31 patients (26.1%); operative intervention (grade 3) had to be performed in 1 patient (0.6%). Five patients (2.2%) developed wound infections: grade 2 wound infections with the need of a local intervention occurred in 3 patients (1.9%) and grade 3 wound infections with the need of antibiotic and operative intervention in 2 patients (1.3%). The incidence for wound dehiscence after surgery was 1.9%: grade 1 in 1 patient (0.6%) and grade 2 in 2 patients (1.3%). No wound dehiscence with the need of surgical intervention and primary wound closure was documented. There were no grade 4 complications after surgery.

Most complications occurring 6–8 weeks after WBI were due to radiotherapy-dependent acute dermatitis grade 1 (75.2%) and grade 2 (15.7%). 4.6% of the patients suffered from dermatitis grade 3. Asymptomatic grade 1 seroma/hematoma was present in 6 patients (3.9%), breast pain grade 1 and 2 in 3 patients (2.0%). 20 patients (13.1%) complained about fatigue after WBI. No grade 4 toxicities were documented. Detailed information about acute toxicities after IOERT and WBI are shown in Tables 3, 4.

**Table 3:** Postoperative toxicity according to CTCAE criteria.

**Postoperative**	**Grade *n* (%)**				
**Total = 157 patients**	**0**	**1**	**2**	**3**	**4**
Seroma/hematoma	110 (70.1)	25 (15.9)	16 (10.2)	1 (0.6)	0
Axillary seroma/hematoma	152 (96.8)	4 (2.5)	0	1 (0.6)	0
Wound infection	152 (96.8)	0	3 (1.9)	2 (1.3)	0
Wound dehiscence	152 (96.8)	1 (0.6)	2 (1.3)	0	0

**Table 4:** Toxicity according to CTCAE criteria 6–8 weeks after WBI.

**6–8 weeks after WBI**	**Grade *n* (%)**				
**Total = 153 patients**	**0**	**1**	**2**	**3**	**4**
Skin (dermatitis)	6 (3.9)	115 (75.2)	24 (15.7)	7 (4.6)	0
Seroma/hematoma	146 (95.4)	6 (3.9)	0	0	0
Pain	149 (97.4)	1 (0.7)	2 (1.3)	0	0
Fatigue	132 (86.3)	16 (10.5)	4 (2.6)	0	0

### Oncological Outcome/Recurrence Patterns

During follow up, 10 patients suffered from a recurrence event (local in-breast recurrence, contralateral breast recurrence or distant metastatic progression).

Two patients (treated by BCS and WBI) developed a contralateral breast recurrence only, without any further disease progression or ipsilateral recurrence. The contralateral breast recurrence rate was 0.7 and 2.8% after 2 and 3 years, respectively. Patients were also treated with BCS and postoperative WBI and to the time of this analysis, a second recurrence event was not documented.

**Figure 2:**
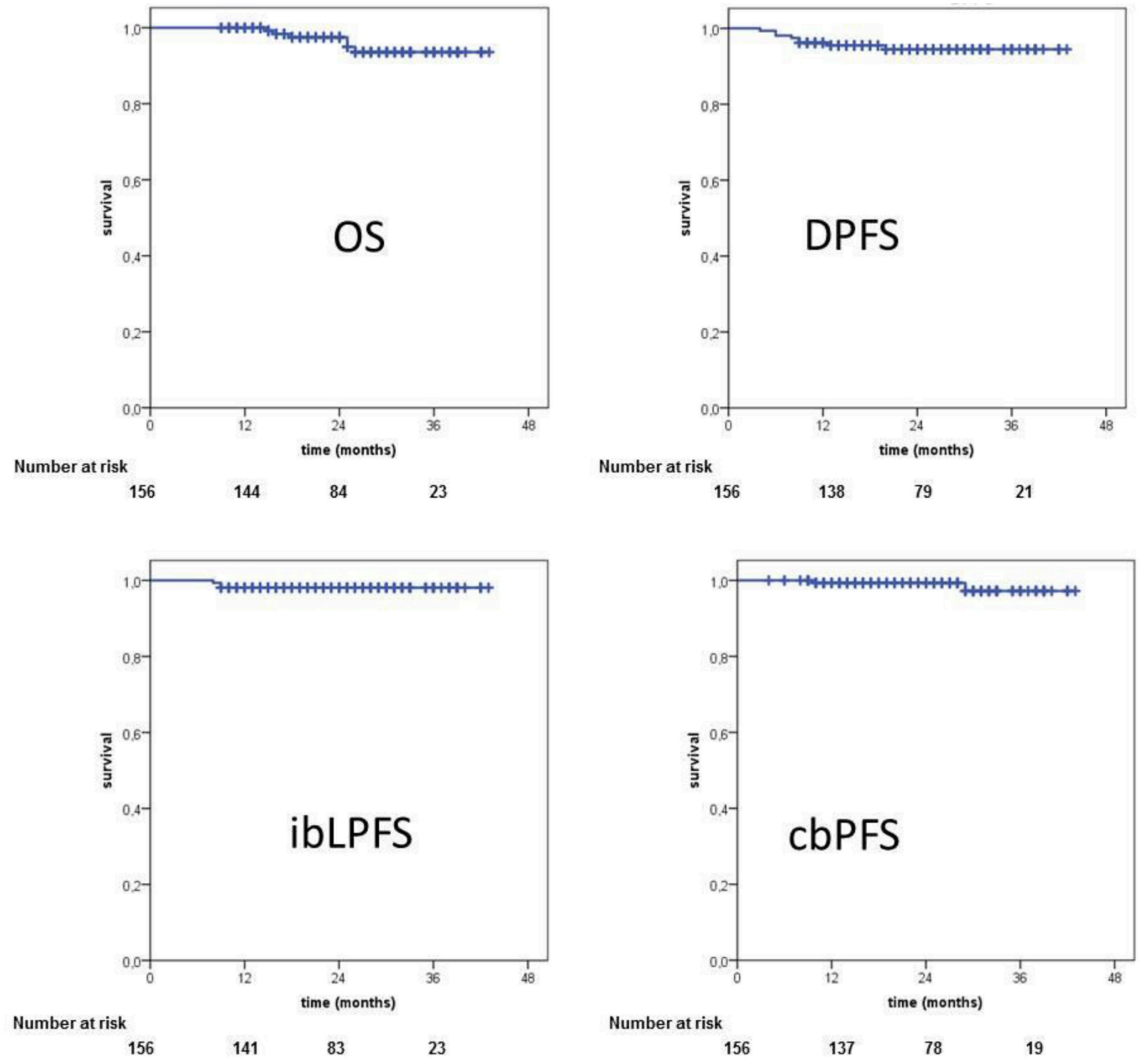
Oncological outcome. OS, overall survival; DPFS, distant progression-free survival; ibLPFS, in-breast local progression-free survival; cbPFS, contralateral breast progression-free survival.

Three patients suffered from an ipsilateral in-breast recurrence, all in-field, leading to a 2- and 3-year in-breast recurrence rate of 1.9% and in-breast local progression-free survival (ibLPFS) of 98.1% (see Figure 2). All 3 patients with local recurrence had triple negative breast cancer (TNBC) and suffered from simultaneous distant disease progression. The in-breast local control is 100% for patients with Luminal A/B and HER2 positive and 80% for triple negative breast cancer patients during the follow up time of our patients.

Distant disease progression with the development of metastasis was documented in 8 patients, leading to a 2- and 3-year distant progression-free survival (DPFS) of 95.5 and 94.5%, respectively (see Figure 2). All patients with distant disease progression displayed aggressive tumor biology (HER2-phenotype, TNBC or G2/G3 grading). Our analysis showed, that median time to progression was short, with only 9.5 months (4–29 months). Table 5 summarizes recurrence patterns of all 10 patients with local and/or distant progression.

**Table 5:** Recurrence patterns.

**Patient**	**Age (years)**	**Death**	**TTP**	**LIBR**	**CBR**	**DMP**	**IP**	**Grading**	**HR status**
1	56–60	No	10	No	Yes	No	Luminal B	2	Positive
2	46–50	No	29	No	Yes	No	Luminal A	1	Positive
3	26–30	Yes	9	Yes	No	Yes	TNBC	3	Negative
4	41–45	Yes	9	Yes	No	Yes	TNBC	3	Negative
5	31–35	Yes	8	Yes	No	Yes	TNBC	3	Negative
6	56–60	No	10	No	No	Yes	Luminal B	3	Positive
7	36–40	No	6	No	No	Yes	Luminal A	2	Positive
8	36–40	Yes	20	No	No	Yes	TNBC	3	Negative
9	61–65	Yes	13	No	No	Yes	HER2	3	Positive
10	51–55	Yes	4	No	No	Yes	TNBC	3	Negative

*TTP, time to progression; LIBR, local in-breast recurrence; CBR, contralateral breast recurrence; DP, distant metastatic progression; IP, immunophenotype; HR, hormone receptor; TNBC, triple negative breast cancer*.

Six patients died during the follow up, yielding in a 2- and 3-year overall survival rate (OS) of 97.5 and 93.6%. Furthermore, all 6 patients had distant disease progression and TNBC or HER2 positive breast cancer. Corresponding Kaplan-Meier charts are displayed in Figure 2.

## Discussion

Primary objective of this analysis was to evaluate acute toxicity after BCS with IOERT as boost radiotherapy for patients with breast cancer treated at our facility. We could demonstrate that IOERT is a safe treatment modality with 97.5% no or only moderate (≤ grade 2) side effects. Grade 3 toxicity with the need of operative intervention occurred only in 4 patients (2.5%). Kaiser et al. (21) also reported only about 3.6% patients with a second operation due to wound complications after IOERT as a boost in 770 patients with breast cancer. Nevertheless, Gülçelik et al. (22) described a higher incidence of seroma, infection and wound complication after IORT compared to surgery alone: Forty-three patients were treated with IORT as a boost and 50 patients with breast-conserving surgery only. After IORT, in 25.5% of the patients a seroma occurred, compared to 6% in the control group. Surgical site infection and delayed wound healing were found in 21 and 35% of the patients compared to 2 and 8% in the control group. These non-randomized data are rather critical. Regarding seroma formation, the rate is consistent with our study. However, 96.8% of our patients showed no wound infection or delayed wound healing and are therefore comparable with their control group. Other authors do not even see a difference in the frequency and amount of seromas after IORT boost (23, 24). In conclusion, IOERT as a boost is a safe and well-tolerated therapy without higher incidence of perioperative complications.

Furthermore, we analyzed oncological outcomes, especially the in-breast recurrence rate. In our study we observed a 2- and 3-year in-breast recurrence rate of 1.9%. The EORTC trial of Bartelink et al. (6) showed a 5-year in-breast recurrence rate of 4.3% after percutaneous boost application without any survival benefit compared to the group without any additional boost. The Salzburg group showed a significant advantage of an intraoperative electron boost in comparison with a percutaneous boost in combination with WBI for patients after BCS. There was an excellent 5-year control rate in the ipsilateral breast with a 0% in-breast recurrence rate compared to 4.3% after percutaneous boost irradiation (25). The same group was able to show excellent local control rates in 770 patients after IOERT boost: After a median follow up time of 121 months, only 2.7% in breast recurrences were observed. Patients with HER2 positive and triple negative breast cancer had a 10-year control rate of 87.9 and 89%, respectively. On the other hand, patients with a Luminal A and B subtype showed an excellent 10-year control rate of 98.7 and 98%, respectively (26). In 2013, the long-term results of a pooled analysis of 6 ISIORT institutions were published. With 1,109 patients this represents the largest published group of patients with IOERT boost in combination with WBI. After a median follow-up time of 72.4 months, only 16 in-breast recurrences were observed yielding a local control rate of 99.2% (9). Regarding the kV-IORT technique a multicenter study revealed similar long-term results. Almost 300 patients were treated with 18–20 Gy at the surface of the applicator with a 5-local control rate of 1.7% (11). Following these results the randomized Targit-B trial is currently ongoing comparing kv-IORT boost with external beam RT boost, especially for women with high risk of local recurrence (ClinicalTrials.gov Identifier: NCT02947425). Taking these results together, the local recurrence could be at least be halved after an intraoperative boost irradiation compared to a percutaneous one. Currently, there are still no randomized data, but all published results showed a favorable trend with intraoperative boost irradiation regarding local control. Possible reasons for an advantage of intraoperative boost irradiation are the opportunity of precise intraoperative visualization of the area with the highest risk for subclinical cell contamination (tumor bed) before the oncoplastic reconstruction takes place. Additionally, due to the high single dose, intra- and interfractional motion is reduced and the biological effectiveness is higher. Perhaps, the improved local control leads to higher patient survival. Data from two non-randomized trials support the hypothesis that local control is a prognostic and predictive factor for distant metastasis or even survival (27, 28). In our study, the 2- and 3-year in-breast recurrence rate is 1.9%, which compares favorably to the other published data with IOERT boost. Interestingly, all patients with a local in-breast recurrence had TNBC and also suffered from distant disease progression with development of metastasis, shortly after the initial diagnosis (median 9 months). Median time to progression in general was short, with only 9.5 months and almost all patients with a recurrence suffered from an aggressive immunophenotype.

Nevertheless, a weakness of this analysis is its retrospective nature and a possible treatment bias, since IORT is only available at a limited number of centers and we did not collect a control group with percutaneous boost application. Certainly a point of criticism in our study is the short follow up time of 24 months in median. The local control can be underestimated by the Kaplan Meier calculation due to the early recurrences of the histologically unfavorable subgroup. At the same time, late recurrences of the more favorable histological subgroup may not be taken into account. Furthermore, conclusions about long term toxicities like cosmesis or fibrosis are not possible.

Despite this, several publications presented an advantage of IOERT boost irradiation in terms of local control. A potential disadvantage of IOERT boost includes the lack of final histopathological reports and the potential need of re-excision in case of positive margins in the specimen. Our analysis could demonstrate that this risk was low with only 11.5% re-excision rate and without higher rates of wound complications due to the previous IOERT. In the ISIORT analysis re-resection was needed in a similar proportion of 10% (9).

## Conclusion

IOERT boost in breast cancer treatment during BCS is a feasible option with low acute toxicity. Despite limitations of our study and the short follow-up period, recurrence rates are comparable to previously published data and emphasize, that IOERT as boost is an effective treatment.

## Ethics Statement

This study is in compliance with the Declaration of Helsinki and was approved by the Independent Ethics Committee Heidelberg (Ref. Nr. S-164/2012).

## Author Contributions

LK and KL contributed equally in data collection, writing, and original draft preparation. KL, LK, SK, MH, SH, TB, JH-R, GM, and MU were responsible for the IOERT procedures as well as radiooncological follow up documentation. MG and JH were the gynecologists responsible for BCS and gynecological follow up documentation of the patient. LK and JH-R performed the statistical analysis. MU and JD conceived of the study and participated in its design and coordination. All the authors were responsible for data interpretation, participated in manuscript revisions, and approved the final manuscript.

### Conflict of Interest Statement

The authors declare that the research was conducted in the absence of any commercial or financial relationships that could be construed as a potential conflict of interest.
